# Genomic investigation of etiologic heterogeneity: methodologic challenges

**DOI:** 10.1186/1471-2288-14-138

**Published:** 2014-12-22

**Authors:** Colin B Begg, Venkatraman E Seshan, Emily C Zabor, Helena Furberg, Arshi Arora, Ronglai Shen, Jodi K Maranchie, Matthew E Nielsen, W Kimryn Rathmell, Sabina Signoretti, Pheroze Tamboli, Jose A Karam, Toni K Choueiri, A Ari Hakimi, James J Hsieh

**Affiliations:** Department of Epidemiology and Biostatistics, Memorial Sloan Kettering Cancer Center, New York, NY USA; Department of Urology, University of Pittsburgh Medical Center, Pittsburgh, PA USA; Departments of Urology, Epidemiology and Health Policy and Management and Surgery, University of North Carolina at Chapel Hill, Chapel Hill, NC USA; Departments of Medicine and Genetics, Lineberger Comprehensive Cancer Center, University of North Carolina, Chapel Hill, NC USA; Department of Pathology, Brigham and Womens’ Hospital, Harvard Medical School, Boston, MA USA; Department of Pathology, The University of Texas MD Anderson Cancer Center, Houston, TX USA; The University of Texas MD Anderson Cancer Center, Houston, TX USA; Department of Medical Oncology, Dana-Farber Cancer Institute, Brigham and Women’s Hospital, Harvard Medical School, Boston, MA USA; Department of Surgery, Urology Service, Memorial Sloan Kettering Cancer Center, New York, NY USA; Human Oncology and Pathogenesis Program, Memorial Sloan Kettering Cancer Center, New York, NY USA

**Keywords:** Etiologic heterogeneity, Kidney cancer, Tumor sub-types

## Abstract

**Background:**

The etiologic heterogeneity of cancer has traditionally been investigated by comparing risk factor frequencies within candidate sub-types, defined for example by histology or by distinct tumor markers of interest. Increasingly tumors are being profiled for molecular features much more extensively. This greatly expands the opportunities for defining distinct sub-types. In this article we describe an exploratory analysis of the etiologic heterogeneity of clear cell kidney cancer. Data are available on the primary known risk factors for kidney cancer, while the tumors are characterized on a genome-wide basis using expression, methylation, copy number and mutational profiles.

**Methods:**

We use a novel clustering strategy to identify sub-types. This is accomplished independently for the expression, methylation and copy number profiles. The goals are to identify tumor sub-types that are etiologically distinct, to identify the risk factors that define specific sub-types, and to endeavor to characterize the key genes that appear to represent the principal features of the distinct sub-types.

**Results:**

The analysis reveals strong evidence that gender represents an important factor that distinguishes disease sub-types. The sub-types defined using expression data and methylation data demonstrate considerable congruence and are also clearly correlated with mutations in important cancer genes. These sub-types are also strongly correlated with survival. The complexity of the data presents many analytical challenges including, prominently, the risk of false discovery.

**Conclusions:**

Genomic profiling of tumors offers the opportunity to identify etiologically distinct sub-types, paving the way for a more refined understanding of cancer etiology.

**Electronic supplementary material:**

The online version of this article (doi:10.1186/1471-2288-14-138) contains supplementary material, which is available to authorized users.

## Background

In the past several years much effort has been expended in identifying tumor sub-types that are clinically distinct, using genome-wide molecular profiling [1]. Most of the attention has been directed at expression arrays, but other profiling tools have also been studied. Investigators have typically “validated” the discoveries by demonstrating that the sub-types possess distinct clinical characteristics, such as case survival. Relatively little attention has been paid to the goal of distinguishing sub-types on the basis of etiology. Etiologic heterogeneity of cancer has traditionally been investigated by comparing risk factor frequencies within candidate sub-types, defined for example by histology or by distinct tumor markers of interest. In kidney cancer several studies have explored the relation of tumors with mutations in the von-Hippel-Lindau (VHL) gene to risk factors such as history of smoking and hypertension [2–4]. More general reviews of research relating risk factors to epigenomic profiles have been compiled recently, though the statistical methodology for establishing etiologically distinct sub-types based on genomic profiles in the presence of multiple risk factors is largely undeveloped [5, 6]. Since more extensive genomic profiling of tumors is likely to become commonplace in the future epidemiologists will be focusing increasing attention on the task of discovering etiologically distinct sub-types, rather than on studies restricted to sub-types defined by specific candidate markers. This exercise will be challenging due to the vast amounts of genomic data available on modern tumor profiling platforms and the consequent risks of false discovery.

In this article we present a prototype discovery analysis using data from The Cancer Genome Atlas (TCGA). We have available several hundred cases from the (clear cell) kidney TCGA for which data have also been assembled on the known risk factors for kidney cancer. We build on an analytic strategy that was developed by members of our team and used previously to study the etiologic heterogeneity of breast cancer [7]. The breast cancer analysis involved data from two large case-control studies [8, 9]. However, the tumors were characterized by only 4 expression markers, and so the capacity to define sub-types was extremely limited. Our present dataset contrasts with this in that the tumors have all been extensively profiled using multiple genomic platforms. Also, our sample is restricted to cases. Thus the present study is extremely rich in tumor profiling, allowing us to evaluate much more completely and rigorously our proposed techniques for identifying etiologically distinct sub-types.

The goals of the article are two-fold. First, we endeavor to demonstrate a novel strategy for identifying etiologically distinct tumor sub-types from extensive tumor profiling data, and to explore the methodological challenges. Second we seek to discover clues about the distinctive etiologies of different types of kidney cancer.

## Methods

We use publically available genomic profiling data generated by the TCGA, together with data on known risk factors for kidney cancer extracted from medical records. The study was approved by the Memorial Sloan Kettering Cancer Center Institutional Review Board/Privacy Board, the University of Pittsburgh Institutional Review Board, the Biomedical IRB of the University of North Carolina, the Dana Farber Cancer Institute Office for Human Research Studies and the MD Anderson Cancer Center Institutional Review Board. The data were obtained from chart review from the source sites of the TCGA in accordance with the individual sites’ IRBs. All patients provided written informed consent for the use of their records. Each participating institution updated the clinical information for its own patients. Some of the authors on the study are clinicians that treated the patients within the TCGA and thus had access to specific patient records. Initial analyses of the kidney TCGA revealed four sub-types identified by clustering the mRNA data that were observed to be characterized by distinctive mutational profiles [10].

### Data

Cases were selected for the TCGA project based on patient consent and the availability of adequate tissue for the intensive planned mutational analyses. Thus the selection of cases cannot be considered representative of all diagnosed cases and may result in a preponderance of features characteristic of more advanced cases with larger tumors or may under-represent metastatic cases that frequently do not undergo nephrectomy.

We elected to focus on four distinct genomic platforms: mRNA, copy number, methylation and mutation. mRNA expression results were generated from the Illumina HiSeq platform. We used normalized log counts and filtered out genes with low expression (median <5 counts) and low variability (MAD < 1.25), following standard practice of TCGA investigations leaving 1267 genes from the original panel of 20531. Methylation data were generated from the Illumina 27k and 450k panels as described previously [8]. A total of 25014 probes were examined, with the sex chromosome excluded. Data were standardized across samples and within platform and merged, and the top 1000 most variable probes selected for analysis. Copy number data were derived from Affymetrix SNP 6.0 arrays. We used a reduction parameter (ϵ = 0.001) to obtain a total of 2312 regions, and our data comprise the segment means from each region. These filtering approaches are based on the premise that about 1000 probes is sufficient to capture any relevant structure in the data, while the addition of more probes, especially those with low signal or low variance, is likely to add noise. Mutation data were obtained from the supplementary files of the original publication of the TCGA without any additional processing [10].

Risk factor data were obtained from the medical records. We obtained information on smoking status at diagnosis (current, former, never smoker), body mass index (BMI) categorized in accordance with World Health Organization criteria (<25, 25-29, 30+ kg/m^2^) and lifetime history of hypertension (yes, no), all of which are established risk factors for kidney cancer [11–14]. In addition we include age and gender, since cancer incidence in general is influenced by both of these factors. Instructions for how to reconstruct the data are provided in Additional file 1 Supplementary Materials (Data Archive).

### Analytic framework

Details of our general analytic strategy were explained in a previous article [7]. In the following we summarize the essential conceptual features of the approach, and some modifications we have made to suit the nature of the TGCA data, namely the extensiveness of the genomic profiling and the fact that the study is limited to cases with cancer but not healthy controls. Our primary goal is to identify tumor sub-types that are etiologically distinct. To accomplish this we use a hybrid clustering strategy that employs classical k-means clustering using the genomic profiles of the tumors to identify candidate solutions. K-means clustering endeavors to find the set of clusters that maximizes the weighted Euclidean distance between the clusters using the inter-cluster dissimilarity, denoted by G, as the distance measure. Because of the complexity of identifying the maximum of a scalar function in multi-dimensional space k-means clustering from an initial random seed inevitably reaches a local maximum rather than the global maximum. Thus the method involves repeated maximization using different random seeds, with the maximum of the various local maxima chosen as the ultimate solution. In our approach, rather than choosing the solution with the highest value of G, for each local maximum we calculate a measure of etiologic heterogeneity and choose the solution with the highest value of this measure. We used 10,000 k-means runs for this purpose. Empirically the individual values of the clustering measure identified (defined below) were each observed sufficiently frequently that we are confident we did not fail to identify the maximum. Each clustering analysis involves initial specification of the number of clusters. That is, we perform an analysis based on the assumption that there exist 2 clusters, then we perform an analysis based on 3 clusters, and so forth.

Our measure of etiologic heterogeneity is based on two related concepts. The first is that in studying risk factors we desire to maximize the predictability of disease occurrence in individuals, and that a useful measure of predictability is the extent of variation of the risks of individuals in the population. That is, the more widely varying the individual risks, the more easily we are able to predict the disease. We use for this purpose the coefficient of variation of disease risks, denoted by K, a measure that aggregates the relative contributions of individual risk factors. In any disease sub-type the corresponding coefficient of variation of the risks of the sub-type is denoted K_j_ for sub-type j. That is, if r_i_ is the overall disease risk for the i^th^ individual and r_ji_ is the corresponding risk of sub-type j, then *Κ* = *v*^1/2^/*μ* and  where  and where n is the number of subjects in the population at risk. The etiologic heterogeneity of sub-types can be characterized by the correlations of the risks of the individual sub-types, with low (or negative) correlation representing high degrees of heterogeneity. Thus the coefficients of covariation, *Κ*_*jk*_ = *c*_*jk*_/*μ*_*j*_*μ*_*k*_, where *c*_*jk*_ = *n*^- 1^∑*r*_*ji*_*r*_*ki*_ - *μ*_*j*_*μ*_*k*_, reflect (inversely) the degrees of etiologic heterogeneity between pairs of sub-types. The second concept is that increasing etiologic heterogeneity between sub-types inevitably increases the collective risk predictability within sub-types. Thus by using a measure of incremental risk prediction denoted by
1

where *π*_1_, *π*_2_, … *π*_*m*_ represent the proportions of cases in each of m sub-types, we are able to choose sets of sub-types that maximize the extent to which the average risk predictability of the set of sub-types (the term in parentheses) exceeds the risk predictability of the disease as a unitary entity (as represented by K^2^), and by so doing we also maximize the collective etiologic heterogeneity of the sub-types. This can be seen by observing that D can also be written in the following way, showing that it increases with decreasing values of the covariances:-
2

where the summation extends to all pairs of sub-types.

To calculate the various coefficients of variation and covariation one needs to obtain risk predictors for each sub-type for each case. In the context of a case-control study these can be obtained from polytomous logistic regression of the sub-types on the risk factors, as described in our previous work [7]. However, the kidney TCGA dataset contains only cases, with no disease-free controls. The case-only design permits estimation of the ratios of the relative risks of the different sub-types for any subject but does not permit estimation of the relative risk of disease itself [15]. However, we can calculate an approximation to D, denoted D^*^, that captures the essential features of the heterogeneity signal as follows.

The preceding formulas (1) and (2) represent averages with respect to the population at risk. Since the controls in a case-control study represent the population at risk the variance and covariance components of the formulas must be estimated by averaging over the controls. In a case-only study we can only calculate such terms using cases, and so corresponding summation terms represent averages over the population distribution of cases. Cases occur based on risk-biased sampling from the population at risk, and so the various terms we use in calculating our measure of etiologic heterogeneity are averaged with respect to this risk biased sample. Risk biased sampling means that individuals become cases in direct proportion to the individual’s risk. Consequently to deconvolute the distribution of risks obtained from a sample of cases in order to equate it with the corresponding distribution from controls one would have to reweight each case in inverse proportion to its risk, i.e. the i^th^ case must be reweighted by the factor *μ*/*r*_*i*_, relative risks that are not estimable in the setting of a case-only study. In the absence of controls we must simply estimate the variance and covariance terms that comprise D using the cases. To see the impact of this we make use of the fact that D can be re-expressed in terms of individual, case-specific deviations of the sub-type probabilities from their overall relative frequencies as follows:-
3

where *u*_*ji*_ =* r*_*ji*_*/r*_*i*_ represents the conditional probability that the i^th^ case belongs to the j^th^ sub-type. The last term in parentheses represents the deviation of the sub-type probabilities for the i^th^ case for the j^th^ and k^th^ sub-types. Greater etiologic heterogeneity is reflected by larger values of these deviations. If we simply use cases to estimate the variances and covariances that comprise D in (1) then we are in effect estimating D^*^, where
4

That is, the contributions of individual cases are additionally weighted in proportion to their risks via the terms {*r*_*i*_/*μ*}. The effect of this change will be to give greater weight in (4) to risk strata with higher risks and correspondingly lesser weight to risk strata with lower risk. We cannot compare these terms empirically since we have no controls, but it is clear that the impact of the difference will be minimal unless there is both a very broad range of individual risks, and a trend for the “outliers” to occur preferentially at one end of the risk scale. Moreover, the goal of our analysis is not to evaluate the absolute magnitude of D. It is to use relative values of D to rank different sub-typing options to determine which ones exhibit the greatest degrees of etiologic heterogeneity. Intuitively the rankings of D and D* are likely to be very similar in practice, even in the presence of broad variation in the underlying risks.

We evaluated the statistical significance of the hypothesis that heterogeneity exists in the data in the following way. We determined the value of D* from the optimal 2-class system and compared this with a reference distribution in which the sample labels were permuted 1000 times and D* recalculated for the new dataset. Permutation of the sample labels ensures that the genomic profiles are randomly paired with the risk factor profiles, defining the absence of a true signal. Determination of the correct number of sub-types is a challenge in any clustering context but it is especially challenging in this context. Here we chose to use the difference in the optimal D* values for the numbers of sub-types being compared, e.g. in determining whether 3 sub-types reveals significant additional heterogeneity to 2 sub-types we subtracted the optimal D* for the 2-class analysis from the optimal value for the 3-class analysis. We generated a reference distribution by permuting the sample labels, calculating the optimal 3-class and 2-class solutions, calculating the difference, and repeating the process 1000 times.

Our investigation is exploratory. Since genomic data are so voluminous and we have results from multiple platforms we approached the analysis with some specific questions in mind, to provide structure to our analysis and to enhance our confidence in any interesting observations. First we performed the preceding clustering analysis separately for each of 3 platforms: mRNA, methylation and copy number arrays. Then we attempted to address the following questions:- Do any of the identified sub-types possess a distinctive risk profile? Are any sub-types determined from mRNA, methylation or copy number data characterized by distinct mutational profiles or genetic pathways? Do the individual sub-types have distinctive clinical characteristics? Are the different genomic platforms congruent with respect to sub-types identified?

To address the involvement of genetic pathways a gene set enrichment analysis was conducted. We obtained a pre-defined collection of pathway gene sets from the Molecular Signatures Database (MSigDB database v4.0) and the database for Annotation, Visualization, and Integrated Discovery (DAVID). We conducted a gene set enrichment analysis for each of the subtypes for each of the platforms. Specifically, for each platform we first calculated a t-statistic for each gene j comparing samples in sub-type k (k = 1,..,4) versus the remaining sub-types. Genes were ranked based on these scores. Then for each gene set S, a Wilcoxon rank sum test was used to compare the ranks of genes in the pathway (j ∈ S) versus their complement (j ∉ S). In this way we calculated a separate enrichment p-value for each pathway in each of the four subtypes. This can be considered a competitive test in the nomenclature of Goeman and Buelmann in that the Wilcoxon test statistic assesses whether the frequency of differential expression differs for pathway genes versus non-pathway genes [16].

## Results

The TCGA dataset comprises 442 cases of clear cell renal cancer. Data on risk factors were retrieved from medical records at Memorial Sloan Kettering Cancer Center, the University of North Carolina, the University of Pennsylvania Medical Center, Dana-Farber Cancer Institute, and MD Anderson Cancer Center for a total of 332 of these cases, and these form the basis for our analyses.

### Distinctiveness of sub-types identified with respect to risk profiles

We conducted analyses for each of the genomic platforms separately. We tested first for the presence of etiologic heterogeneity with both mRNA and methylation profiling exhibiting statistically significant heterogeneity (p < 0.01) while copy number profiling did not (p = 0.11). It has been observed that copy number alterations occur much less frequently in this disease than in other cancers examined in the TCGA project [10]. We then explored the optimal numbers of potential sub-types for mRNA and methylation profiling. The results of our tests are displayed in Table 1. These do not demonstrate a consistent pattern, suggesting around 3-4 sub-types based on mRNA profiling but a larger number of sub-types based on methylation profiling. In the absence of consistency we have elected to present results only for the 4-class solutions. This facilitates comparison of the solutions in different platforms and also with the 4 class solution derived by the TCGA investigators using unsupervised clustering [10].

**Table 1:** **Selecting the optimal number of clusters**

Incremental # sub-types	P-values ^a^	
	mRNA	Methylation
**3 vs 2**	0.02	<0.01
**4 vs 3**	0.06	0.04
**5 vs 4**	0.41	<0.01

^a^The p-values determine whether the designated increase in the numbers of clusters leads to significant additional etiological heterogeneity, as described in the text.

**Figure 1:**
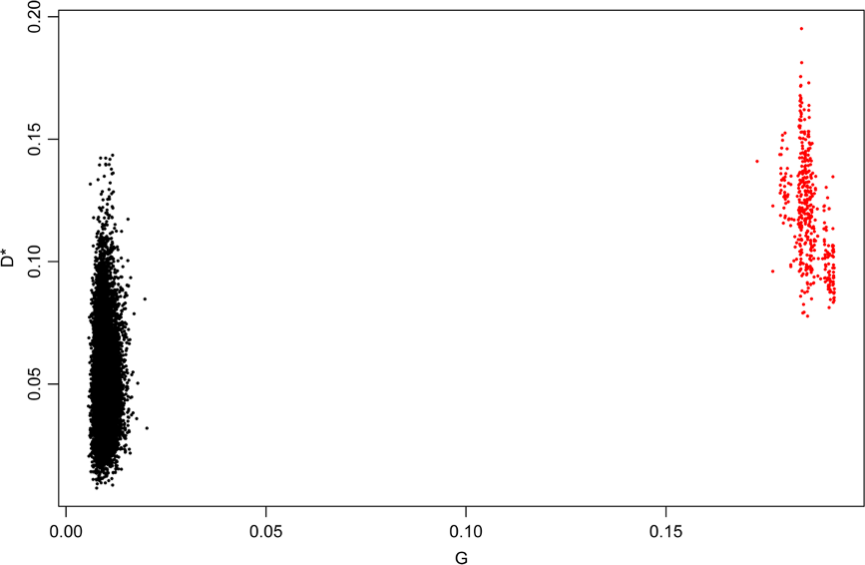
**Distributions of clustering solutions.** The red dots represent local maxima of unsupervised clustering based on G (horizontal axis). The black dots represent solutions based on random permutations of case labels for which no signal on either axis is present.

Consider first the analysis involving mRNA expression data. This analysis is based on 313 cases. We performed the clustering 10,000 times using different random seeds and this led to 533 unique solutions at local maxima. The D^*^ measure of etiologic heterogeneity (Y-axis) and the corresponding distance measure G (X-axis) are plotted in Figure 1 in red for each of these 533 solutions. The black dots represent solutions in which the cases are randomly assigned to 4 sub-types to create datasets in which the sub-types are not associated with the tumor profiles. Thus these black dots benchmark the G values expected when there is no genuine sub-structure to the gene expression profiles. If the gene expression sub-structure is associated with the risk factors then we would expect the D^*^ values corresponding to the red dots to be stochastically larger than those of the black dots as seen in the figure. The “optimal” solution is the highest of the red dots on the vertical axis. By contrast, a standard unsupervised clustering solution would be based on the largest value of G (horizontal axis). Clearly these two solutions are quite far apart in Figure 1, and indeed they represent sets of sub-types with only modest overlap. A cross tabulation of the 4 classes created by these two solutions shows that at most we can align 49% of the cases into congruent classes; the remaining 51% are necessarily incongruent.

In Table 2 we present the distributions of risk factors within each of the sub-types. Since between-class distinctions in these profiles drive the creation of the sub-types we cannot use conventional statistical tests to assess the statistical significance of observed differences. The results seem to show that sub-type 4 has a strong female predominance relative to the other sub-types, and this sub-type is also characterized somewhat by low prevalence of smoking and elevated history of hypertension.

**Table 2:** **Expression sub-types** ^**a**^

Risk factor		Sub-type 1	Sub-type 2	Sub-type 3	Sub-type 4
		n = 74	n = 83	n = 71	n = 85
**Smoking**	**Never**	45%	40%	47%	66%
	**Former**	41%	36%	47%	25%
	**Current**	15%	24%	7%	9%
**BMI**	**Normal**	23%	22%	24%	17%
	**Overweight**	35%	28%	44%	28%
	**Obese**	42%	51%	32%	55%
**Hypertension**	**No**	38%	52%	45%	33%
	**Yes**	62%	48%	55%	67%
**Gender**	**Male**	70%	78%	72%	41%
	**Female**	30%	22%	28%	59%
**Age**	**Median**	62	58	61	63

^a^The entries represent the relative frequencies of the risk factors within sub-types.

We performed an analogous clustering analysis using methylation data. Data were available on 326 cases. We again performed the clustering 10,000 times using different random seeds and this led to 114 unique solutions at local maxima. The distributions of risk factors for the sub-types in the “optimal” solution are displayed in Table 3. The most striking observation is that sub-type 4 has elevated representation of both females and cases with a history of hypertension, similar to the results for mRNA. Again, no apparent differences strongly distinguish the remaining 3 classes. These sub-types have been arbitrarily numbered to identify most closely congruences with the clusters derived using mRNA data, using the 313 cases for which results are available for both mRNA and methylation. Thus sub-type 1 mRNA has the most overlap with sub-type 1 methylation, and so forth.

**Table 3:** **Methylation sub-types** ^**a**^

Risk factor		Sub-type 1	Sub-type 2	Sub-type 3	Sub-type 4
		n = 80	n = 83	n = 70	n = 93
**Smoking**	**Never**	49%	58%	40%	48%
	**Former**	44%	23%	46%	37%
	**Current**	8%	19%	14%	15%
**BMI**	**Normal**	13%	22%	30%	22%
	**Overweight**	41%	25%	33%	34%
	**Obese**	46%	53%	37%	44%
**Hypertension**	**No**	53%	46%	46%	25%
	**Yes**	47%	54%	54%	75%
**Gender**	**Male**	75%	63%	71%	53%
	**Female**	25%	37%	29%	47%
**Age**	**Median**	62	54	61	64

^a^The entries represent the relative frequencies of the risk factors within sub-types.

### Mutational profiles of sub-types

We observed some quite strong associations between the mRNA and methylation sub-types and mutations in selected genes. The data are displayed in Tables 4 and 5, respectively. The most notably distinct mRNA sub-types are sub-type 3, characterized by BAP1, PTEN and TP53 mutations, and sub-type 4, characterized by elevated frequencies of VHL and PBRM1 mutations. Strikingly, the methylation classification sub-type 4 also exhibits high frequency of PBRM1 mutations while sub-type 1 exhibits low frequencies. Similar congruence was seen for methylation sub-type 3 which is also characterized by BAP1 and TP53 mutations, although elevated frequency of PTEN mutations was not observed.

**Table 4:** **Mutations in mRNA sub-types** ^**a**^

Gene	# Cases	Sub-type 1	Sub-type 2	Sub-type 3	Sub-type 4	p-value
		n = 74	n = 83	n = 71	n = 85	
**VHL**	162	58%	54%	39%	69%	.003
**PBRM1**	97	32%	39%	12%	46%	<.001
**SETD2**	36	10%	14%	12%	12%	.90
**BAP1**	33	13%	6%	24%	5%	.001
**MTOR**	22	6%	9%	7%	9%	.90
**ADAM6**	22	4%	6%	4%	14%	.07
**MST1P2**	22	6%	10%	6%	8%	.76
**PDE4DIP**	18	7%	3%	6%	9%	.48
**KDM5C**	17	10%	6%	4%	3%	.24
**PTEN**	12	6%	1%	10%	0%	.009
**TP53**	8	1%	1%	9%	0%	.006

^a^The entries represent the frequencies of occurrence of mutations in the given genes.

**Table 5:** **Mutations in methylation sub-types** ^**a**^

Gene	# Cases	Sub-type 1	Sub-type 2	Sub-type 3	Sub-type 4	p-value
		n = 80	n = 83	n = 70	n = 93	
**VHL**	162	48%	61%	45%	64%	.04
**PBRM1**	97	12%	31%	31%	55%	<.001
**SETD2**	36	5%	6%	23%	15%	.003
**BAP1**	33	11%	4%	25%	7%	<.001
**MTOR**	22	8%	6%	6%	10%	.80
**ADAM6**	22	5%	7%	5%	12%	.30
**MST1P2**	22	9%	10%	8%	4%	.41
**PDE4DIP**	18	7%	6%	6%	6%	1.00
**KDM5C**	17	7%	3%	5%	8%	.50
**PTEN**	12	8%	0%	5%	4%	.11
**TP53**	8	4%	0%	8%	1%	.04

^a^The entries represent the frequencies of occurrence of mutations in the given genes.

Gene set enrichment analysis revealed that the solute carriers (SLC) transporter gene family was the pathway most differentially expressed in mRNA sub-type 4 (although it did not exceed the Bonferroni correction for multiple testing). Many SLCs are involved in metabolism and kidney cancer has been characterized as a metabolic disease [17]. Changes in transporter expression can affect the movement of drugs and their metabolites across cell membranes and thus impact drug sensitivity [18]. SLC-transporter expression has been associated with chemosensitivity in various cancer types including kidney cancer [19, 20]. Such an expression difference observed for the SLC gene family may be potentially important to explain differences in tumor biology and may have treatment implications for this female-predominant expression sub-type. The most significant pathway affecting methylation sub-type 4 was transcription regulation, suggesting that expression changes associated with this subtype may be methylation-driven. Indeed, several SLC family genes including SLC16A5 and SLC13A1 show negative association between methylation and gene expression.

### Clinical characteristics of sub-types

**Figure 2:**
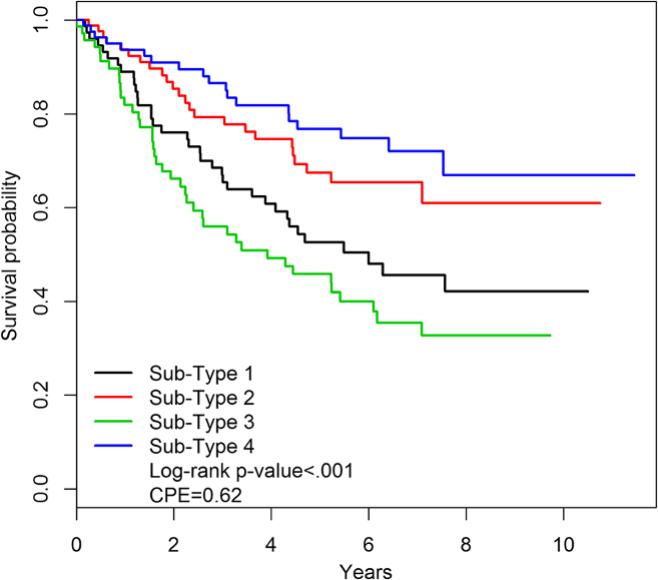
**Survival of mRNA sub-types.** Kaplan-Meier survival curves for cases classified in 4 mRNA sub-types.

**Figure 3:**
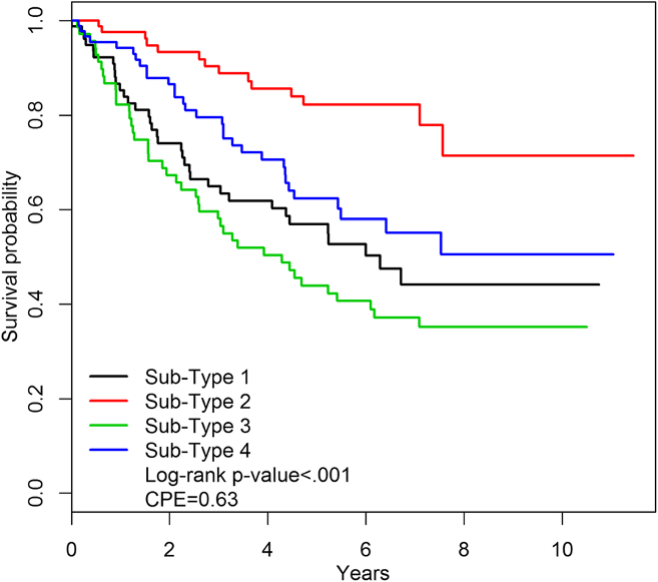
**Survival of methylation sub-types.** Kaplan-Meier survival curves for cases classified in 4 methylation sub-types.

Conventional methods for determining tumor sub-types, as employed by the TCGA investigators and many other groups, involve the use of unsupervised clustering, and validation of the biologic significance of the sub-types is in part determined by whether the sub-types display distinct clinical characteristics such as distinctive distribution of histology, stage or survival. The TCGA sub-types obtained using unsupervised clustering demonstrate quite substantial and highly significant differences in survival [10]. The c-index associated with the TCGA mRNA sub-types is 0.63. However, the mRNA sub-types determined on the basis of etiologic heterogeneity also demonstrate strong and highly significant survival differences with a similar c-index of 0.62 (Figure 2). Likewise the methylation sub-types display strong and significant separation on the basis of survival with a c-index of 0.63 (Figure 3). In short, our method succeeded in obtaining sub-types optimally clustered on the basis of etiologic heterogeneity without apparently sacrificing any association with survival. Note that these comparisons are appropriately unadjusted for prognostic factors such as stage since the goal is to see if the sub-types are clinically distinctive in an absolute sense.

### Congruence of sub-types across platforms

Ideally the analyses using different platforms would suggest similar sub-types, giving us confidence that the clustering is producing verifiable sub-types. The numbers of cases that are congruent for mRNA and methylation sub-types 1, 2, 3 and 4 are, respectively, 26, 39, 39 and 48, leading to an overall congruence of 152 (49%) of the cases, versus 79 (25%) expected if the categories were generated randomly (p < 0.001). Of these the most credible overlap of sub-types with similar risk factor profiles is between sub-type #4 for both mRNA and methylation. Both of these sub-types have a strong female predominance, and also the suggestion of a common hypertension predominance. Sub-type 3 in both platforms is characterized by BAP1 and TP53 mutations.

## Discussion

Previous investigations of the molecular epidemiology of kidney cancer have focused attention on cases classified on the basis of mutations in the VHL gene which has been recognized for many years as a source of common early mutations in the development of this disease [21, 22]. A study by van Dijk et al. [2] concluded that smoking is unrelated to VHL mutations but a later study by Moore et al. [4] appears to contradict this finding. Schouten et al. [3] provide evidence that hypertension is positively associated with the occurrence of VHL mutant cases while diuretic use is associated with VHL negative tumors. However, in all of these studies the associations observed are modest. Our approach has been to address the molecular epidemiological associations in an exploratory fashion using a much larger compilation of genetic markers.

We have presented a prototype investigation of this strategy, making use of the availability of risk factor data on a series of cases that have been extensively profiled as part of the TCGA project. We anticipate that data on extensive tumor profiling will become increasingly available in case-control and other epidemiologic investigations, and so we have outlined some options for approaching the analysis of these kinds of study. Because of the vast quantities of genomic data the analysis is inevitably built around the concept of clustering, a statistical strategy designed to identify groups of cases that are “similar”, and thus may represent distinct disease sub-types. A major concern in all clustering analyses is the strong possibility of false discovery, and this is certainly a possibility in our study. To convince ourselves that sub-types identified from clustering techniques are meaningful, the ideal validation would be a repeat study in which similar clusters emerge. In the absence of a replicate study we judge the believability of our results using somewhat heuristic strategies. One of these is to examine whether the sub-types are clinically distinctive. On this basis our survival analyses are reassuring, in that they demonstrate strong and statistically significant differences between the sub-types. Another approach we have examined is to see if sub-types determined by different genomic platforms are congruent. There is some evidence that two of the classes identified independently by mRNA and methylation profiling demonstrate considerable concordance, but the evidence here appears suggestive rather than conclusive. We also examined the mutational profiles of the sub-types created from mRNA and methylation profiling, and here the evidence is stronger, with mutation frequencies in some genes strongly and significantly associated with individual sub-types. Collectively, these facts give us some confidence that the sub-types identified represent real classes with distinctive biological and clinical characteristics.

Our major finding that appears supported by strong evidence is the identification of a sub-type that has an elevated female representation compared to other sub-types. This is a clear and striking result that emerges independently from both the mRNA and the methylation profiling. The importance of gender in this disease has been suggested by others, since gender is known to affect both incidence and survival [23]. Recently Brannon et al. have integrated data from multiple genomic studies and have observed that one of the major sub-types clearly segregates along gender lines [24]. Additionally, this sub-type seems to be characterized by mutations in the PBRM1 gene which is located close to the VHL gene in the 3p region [25]. Another sub-type that emerges from both mRNA and methylation profiling appears to be characterized by mutations in BAP1 and TP53.

Our study has limitations. The sample size was limited by the numbers collected for the TCGA project and as such is modest for the ambitious goals of identifying what may actually be many sub-types. Much larger sample sizes are necessary to confidently identify sub-types, especially sub-types with lower frequencies of occurrence. As in all analyses of voluminous genomic data many arbitrary decisions need to be made prior to conducting the analyses. These include, for example, pre-processing decisions that affect the number of probes included in the analysis, the arbitrary exclusion of solutions that contain sub-types with very low numbers of cases, and others. These arbitrary decisions could affect the conclusions. Risk factor data were abstracted from medical records. Smoking status is based on self-report, pre-surgical BMI may be impacted by disease-related weight loss, and history of hypertension does not reflect duration or management of the disease. Hormonal and reproductive factors for women were not available. The absence of controls requires us to use a slightly modified version of the measure of heterogeneity that we proposed in previous work in the context of case-control data. It has been shown that in a case-control setting essentially all of the relevant information concerning etiologic heterogeneity is contained in the “case” information, since it is contrasts in the risk profiles of cases that characterize etiologic heterogeneity [26]. Consequently the use of our modified measure should have minimal, if any, impact on the results that would have been obtained if controls had been available. Finally determination of the correct number of sub-types is challenging. Our statistical test for determining if the addition of an extra sub-type significantly increases the observed heterogeneity signal used a reference distribution in which the differences in the optimal values of D* for the competing numbers of clusters were calculated repeatedly from datasets in which the subject labels were permuted. We constructed the test this way because it is not evident how to assess the null increment in D* beyond the optimal D* observed for the lower number of sub-types. Further research is needed to clarify the operating characteristics of our approach and possibly refine it. More importantly, there is a true underlying set of sub-types, and the fact that the different platforms were not congruent with respect to the numbers of sub-types identified by our testing algorithm demonstrates considerable uncertainty in the data regarding the true number of sub-types.

## Conclusions

Our study is a demonstration of a novel analysis of etiologic heterogeneity taking advantage of the abundant genomic resources available from the TCGA project. Due to a limited sample size the results are necessarily speculative. Our primary observation is that there exists a distinctive sub-type characterized by female gender, and also by PBRM1 mutations. We also observed that the sub-types identified by mRNA and methylation profiling have significantly distinct survival. These results require validation in subsequent investigations.

## Electronic supplementary material

Additional file 1: **Supplementary Materials.** (DOCX 35 KB)

## Abbreviations

VHL: Von-Hippel-Lindau
TCGA: The Cancer Genome Atlas
BMI: Body mass index
SLC: Solute carriers.
