# Supplementary material for: Genomic investigation of etiologic heterogeneity: methodologic challenges
Source: BMC Med Res Methodol. 2014 Dec 22;14:138. doi: 10.1186/1471-2288-14-138 (PMC4292824; doi:10.1186/1471-2288-14-138)
Supplement: Supplementary file 1 — Additional file 1: Supplementary Materials. (DOCX 35 KB) [file 12874_2014_1148_MOESM1_ESM.docx]

**Additional File 1. Supplementary Materials**

**Data Archive**

The genomic data used in the analyses were obtained from the TCGA data portal as follows. The various data elements of clear cell kidney cancer available for download are in the following website:-

<https://tcga-data.nci.nih.gov/tcga/dataAccessMatrix.htm?mode=ApplyFilter&showMatrix=true&diseaseType=KIRC&tumorNormal=TN&tumorNormal=T&tumorNormal=NT>

The top row gives the data type (clinical, exp-gene, etc.), the second row gives the platform (UNC AgilentG4502A_07) and the third row is the data tier (level). We chose the level 3 data for HumanMethylation27 and HumanMethylation450 (two methylation platforms used on nearly mutually exclusive subsets of patients), Genome_Wide_SNP_6 (segmented copy number data using Affymetrix SNP 6 arrays) and IlluminaHiSeq_RNASeqV2 (gene expression summarized as RSEM). These are columns 8, 11, 14 and 24 in the website. Highlighting them and clicking on the “Build Archive” button will generate the necessary files. These data are returned as separate files for each patient and data type which are then processed to obtain the data matrices in the analyses.

For methylation, each sample can have either one (tumor alone) or two files (tumor and tissue normal). We used only the tumor data in the analysis. For copy number, there are 4 segmentation files corresponding to genomic position mapped to hg18 and hg19 builds and full data and germline CNV subtracted data. We used the germline subtracted data mapped to hg19. For mRNA, the RNASeqV2 quantifications gave 6 files for each sample. We used the RSEM normalized results at the gene level.

The mutation data and other patient characteristics were obtained from the supplementary data included in the TCGA paper (reference #10 in the main text of this article). The file “Data_file_S10_KIRC-BCM-BI-UCSC-gapfill-v1.11.txt” contains all the somatic mutations detected in all the samples. The survival data are in “Data_file_S2_clinical_dataset.xlsx”.

The risk factor data are in the attached Table S1. Smoking was coded as 0 (never), 1 (former) and 2 (current). BMI was coded as 0 (<25), 1 (25-30) and 2 (>30). Hypertension was coded as 0 (no) and 1 (yes).

**Table S1.**

| **ID** | **Age** | **Gender** | **Smoking** | **BMI** | **Hypertension** |
| --- | --- | --- | --- | --- | --- |
| TCGA-CZ-4857 | 56 | MALE | 0 | 1 | No |
| TCGA-CZ-4858 | 39 | MALE | 0 | 2 | No |
| TCGA-CZ-4860 | 61 | MALE | 1 | 1 | No |
| TCGA-CZ-4861 | 63 | MALE | 1 | 1 | Yes |
| TCGA-CZ-4863 | 51 | FEMALE | 0 | 1 | No |
| TCGA-CZ-4864 | 86 | MALE | 0 | 0 | Yes |
| TCGA-CZ-4865 | 70 | FEMALE | 1 | 0 | No |
| TCGA-CZ-5454 | 63 | MALE | 1 | 1 | No |
| TCGA-CZ-5455 | 63 | MALE | 0 | 1 | No |
| TCGA-CZ-5458 | 43 | MALE | 0 | 1 | No |
| TCGA-CZ-5460 | 55 | MALE | 0 | 1 | Yes |
| TCGA-CZ-5461 | 52 | MALE | 1 | 2 | No |
| TCGA-CZ-5462 | 83 | MALE | 0 | 1 | Yes |
| TCGA-CZ-5468 | 84 | MALE | 1 | 0 | Yes |
| TCGA-CZ-5469 | 41 | MALE | 0 | 2 | No |
| TCGA-CZ-5987 | 60 | MALE | 1 | 2 | Yes |
| TCGA-CZ-5989 | 60 | MALE | 0 | 1 | No |
| TCGA-CJ-4634 | 60 | FEMALE | 0 | 2 | Yes |
| TCGA-CJ-4635 | 48 | MALE | 1 | 2 | Yes |
| TCGA-CJ-4636 | 51 | MALE | 1 | 2 | Yes |
| TCGA-CJ-4637 | 52 | FEMALE | 0 | 0 | No |
| TCGA-CJ-4638 | 46 | FEMALE | 2 | 0 | No |
| TCGA-CJ-4639 | 49 | FEMALE | 0 | 2 | Yes |
| TCGA-CJ-4640 | 49 | MALE | 1 | 1 | No |
| TCGA-CJ-4641 | 55 | FEMALE | 2 | 2 | Yes |
| TCGA-CJ-4642 | 48 | MALE | 2 | 1 | No |
| TCGA-CJ-4643 | 67 | FEMALE | 0 | 2 | Yes |
| TCGA-CJ-4644 | 48 | FEMALE | 0 | 0 | Yes |
| TCGA-CJ-4868 | 42 | MALE | 0 | 0 | Yes |
| TCGA-CJ-4869 | 49 | MALE | 0 | 1 | No |
| TCGA-CJ-4870 | 58 | FEMALE | 0 | 2 | Yes |
| TCGA-CJ-4871 | 63 | MALE | 0 | 2 | Yes |
| TCGA-CJ-4872 | 51 | MALE | 2 | 2 | Yes |
| TCGA-CJ-4873 | 85 | FEMALE | 0 | 0 | Yes |
| TCGA-CJ-4874 | 73 | FEMALE | 0 | 1 | Yes |
| TCGA-CJ-4875 | 67 | MALE | 1 | 0 | No |
| TCGA-CJ-4876 | 57 | MALE | 0 | 1 | No |
| TCGA-CJ-4878 | 71 | FEMALE | 0 | 2 | No |
| TCGA-CJ-4881 | 41 | MALE | 2 | 1 | No |
| TCGA-CJ-4882 | 57 | MALE | 1 | 1 | Yes |
| TCGA-CJ-4884 | 72 | FEMALE | 0 | 2 | No |
| TCGA-CJ-4885 | 64 | MALE | 1 | 1 | Yes |
| TCGA-CJ-4886 | 42 | FEMALE | 0 | 1 | No |
| TCGA-CJ-4887 | 48 | MALE | 0 | 1 | No |
| TCGA-CJ-4888 | 59 | MALE | 0 | 1 | No |
| TCGA-CJ-4889 | 63 | FEMALE | 0 | 2 | No |
| TCGA-CJ-4890 | 72 | MALE | 0 | 1 | Yes |
| TCGA-CJ-4891 | 57 | FEMALE | 2 | 0 | No |
| TCGA-CJ-4892 | 65 | FEMALE | 0 | 2 | Yes |
| TCGA-CJ-4893 | 76 | FEMALE | 0 | 0 | No |
| TCGA-CJ-4894 | 58 | MALE | 2 | 2 | Yes |
| TCGA-CJ-4895 | 62 | MALE | 0 | 0 | Yes |
| TCGA-CJ-4897 | 79 | FEMALE | 0 | 0 | No |
| TCGA-CJ-4899 | 42 | MALE | 0 | 0 | No |
| TCGA-CJ-4900 | 70 | FEMALE | 0 | 0 | No |
| TCGA-CJ-4902 | 61 | MALE | 0 | 2 | Yes |
| TCGA-CJ-4903 | 50 | MALE | 2 | 1 | Yes |
| TCGA-CJ-4904 | 60 | FEMALE | 0 | 1 | No |
| TCGA-CJ-4905 | 62 | FEMALE | 1 | 2 | Yes |
| TCGA-CJ-4907 | 58 | MALE | 0 | 0 | No |
| TCGA-CJ-4908 | 38 | MALE | 0 | 2 | No |
| TCGA-CJ-4912 | 61 | MALE | 0 | 1 | No |
| TCGA-CJ-4913 | 45 | FEMALE | 0 | 0 | No |
| TCGA-CJ-4916 | 69 | FEMALE | 0 | 2 | Yes |
| TCGA-CJ-4918 | 64 | MALE | 1 | 1 | No |
| TCGA-CJ-4920 | 64 | FEMALE | 2 | 1 | Yes |
| TCGA-CJ-4923 | 63 | MALE | 0 | 1 | Yes |
| TCGA-CJ-5671 | 51 | MALE | 0 | 1 | Yes |
| TCGA-CJ-5672 | 84 | MALE | 0 | 1 | No |
| TCGA-CJ-5675 | 70 | MALE | 0 | 2 | Yes |
| TCGA-CJ-5676 | 47 | MALE | 0 | 2 | No |
| TCGA-CJ-5677 | 54 | FEMALE | 0 | 1 | Yes |
| TCGA-CJ-5678 | 62 | MALE | 1 | 1 | No |
| TCGA-CJ-5679 | 73 | MALE | 1 | 0 | No |
| TCGA-CJ-5680 | 65 | FEMALE | 0 | 1 | Yes |
| TCGA-CJ-5681 | 44 | FEMALE | 1 | 1 | No |
| TCGA-CJ-5682 | 60 | MALE | 1 | 1 | Yes |
| TCGA-CJ-5683 | 78 | MALE | 0 | 2 | Yes |
| TCGA-CJ-5684 | 61 | MALE | 0 | 1 | No |
| TCGA-CJ-5686 | 59 | FEMALE | 1 | 1 | Yes |
| TCGA-CJ-5689 | 90 | MALE | 2 | 0 | Yes |
| TCGA-CJ-6027 | 77 | MALE | 0 | 1 | No |
| TCGA-CJ-6028 | 58 | MALE | 1 | 1 | Yes |
| TCGA-CJ-6030 | 65 | MALE | 1 | 2 | Yes |
| TCGA-CJ-6031 | 54 | MALE | 0 | 2 | No |
| TCGA-CJ-6032 | 63 | FEMALE | 1 | 1 | No |
| TCGA-CJ-6033 | 54 | FEMALE | 1 | 0 | No |
| TCGA-BP-4158 | 69 | MALE | 0 | 2 | No |
| TCGA-BP-4159 | 70 | MALE | 0 | 0 | Yes |
| TCGA-BP-4160 | 67 | MALE | 0 | 2 | No |
| TCGA-BP-4161 | 74 | MALE | 1 | 1 | No |
| TCGA-BP-4162 | 65 | FEMALE | 1 | 2 | Yes |
| TCGA-BP-4163 | 60 | FEMALE | 1 | 2 | Yes |
| TCGA-BP-4164 | 51 | FEMALE | 0 | 0 | Yes |
| TCGA-BP-4165 | 64 | FEMALE | 0 | 2 | No |
| TCGA-BP-4166 | 69 | MALE | 1 | 2 | No |
| TCGA-BP-4167 | 59 | MALE | 1 | 1 | No |
| TCGA-BP-4169 | 76 | FEMALE | 1 | 1 | No |
| TCGA-BP-4170 | 72 | FEMALE | 0 | 1 | No |
| TCGA-BP-4173 | 47 | MALE | 1 | 2 | No |
| TCGA-BP-4174 | 49 | MALE | 1 | 2 | Yes |
| TCGA-BP-4176 | 64 | MALE | 1 | 1 | Yes |
| TCGA-BP-4177 | 66 | MALE | 0 | 0 | No |
| TCGA-BP-4325 | 64 | FEMALE | 0 | 2 | Yes |
| TCGA-BP-4326 | 53 | FEMALE | 0 | 2 | Yes |
| TCGA-BP-4327 | 75 | FEMALE | 1 | 1 | Yes |
| TCGA-BP-4329 | 75 | MALE | 0 | 0 | Yes |
| TCGA-BP-4330 | 60 | FEMALE | 0 | 2 | Yes |
| TCGA-BP-4331 | 52 | MALE | 1 | 2 | No |
| TCGA-BP-4332 | 36 | MALE | 1 | 1 | No |
| TCGA-BP-4334 | 56 | MALE | 1 | 2 | No |
| TCGA-BP-4335 | 65 | FEMALE | 0 | 0 | Yes |
| TCGA-BP-4337 | 76 | FEMALE | 1 | 0 | No |
| TCGA-BP-4338 | 43 | MALE | 0 | 2 | Yes |
| TCGA-BP-4340 | 70 | FEMALE | 1 | 2 | Yes |
| TCGA-BP-4341 | 67 | MALE | 1 | 2 | Yes |
| TCGA-BP-4342 | 79 | MALE | 1 | 2 | No |
| TCGA-BP-4343 | 64 | MALE | 1 | 1 | Yes |
| TCGA-BP-4344 | 76 | FEMALE | 0 | 2 | Yes |
| TCGA-BP-4345 | 62 | MALE | 0 | 1 | No |
| TCGA-BP-4346 | 57 | MALE | 1 | 2 | Yes |
| TCGA-BP-4347 | 74 | MALE | 0 | 2 | No |
| TCGA-BP-4349 | 68 | FEMALE | 1 | 2 | Yes |
| TCGA-BP-4351 | 51 | FEMALE | 0 | 2 | No |
| TCGA-BP-4352 | 74 | FEMALE | 1 | 0 | Yes |
| TCGA-BP-4353 | 61 | MALE | 1 | 2 | No |
| TCGA-BP-4354 | 40 | MALE | 0 | 0 | No |
| TCGA-BP-4355 | 59 | FEMALE | 0 | 1 | Yes |
| TCGA-BP-4756 | 62 | FEMALE | 0 | 0 | Yes |
| TCGA-BP-4758 | 40 | MALE | 0 | 1 | No |
| TCGA-BP-4759 | 50 | MALE | 1 | 2 | Yes |
| TCGA-BP-4760 | 69 | MALE | 2 | 2 | Yes |
| TCGA-BP-4761 | 57 | MALE | 2 | 1 | No |
| TCGA-BP-4762 | 42 | MALE | 0 | 1 | No |
| TCGA-BP-4763 | 79 | FEMALE | 0 | 0 | Yes |
| TCGA-BP-4765 | 43 | MALE | 0 | 1 | No |
| TCGA-BP-4766 | 43 | FEMALE | 0 | 0 | No |
| TCGA-BP-4768 | 72 | FEMALE | 1 | 0 | No |
| TCGA-BP-4769 | 64 | MALE | 0 | 2 | Yes |
| TCGA-BP-4770 | 73 | FEMALE | 1 | 0 | Yes |
| TCGA-BP-4771 | 62 | MALE | 1 | 1 | No |
| TCGA-BP-4774 | 57 | FEMALE | 0 | 2 | Yes |
| TCGA-BP-4775 | 55 | FEMALE | 0 | 2 | Yes |
| TCGA-BP-4776 | 52 | MALE | 0 | 0 | Yes |
| TCGA-BP-4777 | 46 | MALE | 0 | 2 | No |
| TCGA-BP-4781 | 78 | MALE | 1 | 2 | Yes |
| TCGA-BP-4782 | 55 | FEMALE | 2 | 1 | Yes |
| TCGA-BP-4784 | 67 | FEMALE | 1 | 2 | Yes |
| TCGA-BP-4787 | 59 | FEMALE | 0 | 0 | No |
| TCGA-BP-4789 | 48 | MALE | 1 | 1 | No |
| TCGA-BP-4790 | 76 | MALE | 2 | 0 | No |
| TCGA-BP-4795 | 74 | FEMALE | 2 | 0 | Yes |
| TCGA-BP-4797 | 34 | MALE | 0 | 0 | No |
| TCGA-BP-4798 | 74 | MALE | 1 | 0 | Yes |
| TCGA-BP-4799 | 70 | MALE | 1 | 2 | Yes |
| TCGA-BP-4801 | 57 | MALE | 2 | 0 | Yes |
| TCGA-BP-4803 | 79 | MALE | 0 | 2 | Yes |
| TCGA-BP-4804 | 59 | MALE | 1 | 1 | Yes |
| TCGA-BP-4807 | 42 | MALE | 2 | 2 | No |
| TCGA-BP-4959 | 49 | MALE | 2 | 2 | Yes |
| TCGA-BP-4960 | 46 | MALE | 0 | 1 | No |
| TCGA-BP-4961 | 47 | MALE | 1 | 2 | No |
| TCGA-BP-4962 | 58 | MALE | 0 | 2 | No |
| TCGA-BP-4963 | 63 | MALE | 1 | 0 | No |
| TCGA-BP-4964 | 54 | FEMALE | 2 | 2 | No |
| TCGA-BP-4965 | 47 | MALE | 0 | 2 | Yes |
| TCGA-BP-4967 | 76 | MALE | 1 | 2 | Yes |
| TCGA-BP-4968 | 40 | MALE | 1 | 1 | No |
| TCGA-BP-4969 | 63 | FEMALE | 1 | 1 | Yes |
| TCGA-BP-4970 | 44 | MALE | 0 | 0 | No |
| TCGA-BP-4971 | 40 | MALE | 0 | 2 | No |
| TCGA-BP-4972 | 43 | FEMALE | 2 | 1 | No |
| TCGA-BP-4973 | 47 | MALE | 1 | 1 | Yes |
| TCGA-BP-4974 | 58 | MALE | 2 | 0 | Yes |
| TCGA-BP-4975 | 40 | MALE | 2 | 2 | No |
| TCGA-BP-4976 | 77 | MALE | 0 | 2 | No |
| TCGA-BP-4977 | 57 | MALE | 2 | 2 | No |
| TCGA-BP-4981 | 75 | FEMALE | 0 | 0 | Yes |
| TCGA-BP-4982 | 42 | MALE | 0 | 2 | Yes |
| TCGA-BP-4983 | 67 | FEMALE | 2 | 0 | Yes |
| TCGA-BP-4985 | 72 | MALE | 1 | 1 | Yes |
| TCGA-BP-4986 | 75 | MALE | 0 | 1 | Yes |
| TCGA-BP-4987 | 41 | FEMALE | 0 | 0 | No |
| TCGA-BP-4988 | 72 | MALE | 1 | 1 | No |
| TCGA-BP-4989 | 58 | MALE | 2 | 0 | Yes |
| TCGA-BP-4991 | 54 | MALE | 1 | 2 | Yes |
| TCGA-BP-4992 | 66 | MALE | 1 | 2 | Yes |
| TCGA-BP-4993 | 58 | MALE | 0 | 2 | Yes |
| TCGA-BP-4994 | 55 | MALE | 2 | 1 | No |
| TCGA-BP-4995 | 68 | MALE | 1 | 1 | Yes |
| TCGA-BP-4998 | 49 | MALE | 0 | 0 | No |
| TCGA-BP-4999 | 56 | MALE | 0 | 2 | Yes |
| TCGA-BP-5000 | 40 | MALE | 1 | 2 | No |
| TCGA-BP-5001 | 43 | FEMALE | 0 | 2 | No |
| TCGA-BP-5004 | 53 | MALE | 0 | 2 | Yes |
| TCGA-BP-5006 | 61 | MALE | 0 | 1 | Yes |
| TCGA-BP-5007 | 45 | MALE | 0 | 2 | No |
| TCGA-BP-5008 | 46 | MALE | 1 | 2 | No |
| TCGA-BP-5009 | 52 | MALE | 0 | 0 | No |
| TCGA-BP-5010 | 63 | MALE | 1 | 1 | No |
| TCGA-BP-5168 | 75 | MALE | 1 | 2 | Yes |
| TCGA-BP-5169 | 70 | MALE | 1 | 2 | Yes |
| TCGA-BP-5170 | 55 | MALE | 0 | 2 | Yes |
| TCGA-BP-5173 | 75 | MALE | 1 | 1 | Yes |
| TCGA-BP-5174 | 45 | FEMALE | 1 | 1 | No |
| TCGA-BP-5175 | 60 | MALE | 1 | 1 | No |
| TCGA-BP-5176 | 78 | FEMALE | 0 | 2 | Yes |
| TCGA-BP-5177 | 46 | FEMALE | 2 | 1 | No |
| TCGA-BP-5178 | 71 | MALE | 1 | 0 | Yes |
| TCGA-BP-5180 | 53 | MALE | 0 | 2 | No |
| TCGA-BP-5181 | 59 | FEMALE | 1 | 2 | Yes |
| TCGA-BP-5182 | 56 | MALE | 2 | 2 | Yes |
| TCGA-BP-5183 | 57 | MALE | 1 | 2 | No |
| TCGA-BP-5184 | 54 | MALE | 1 | 1 | Yes |
| TCGA-BP-5185 | 56 | MALE | 1 | 2 | Yes |
| TCGA-BP-5186 | 50 | FEMALE | 0 | 0 | Yes |
| TCGA-BP-5187 | 54 | MALE | 0 | 1 | Yes |
| TCGA-BP-5189 | 60 | MALE | 1 | 2 | No |
| TCGA-BP-5190 | 61 | MALE | 0 | 2 | Yes |
| TCGA-BP-5191 | 79 | MALE | 1 | 1 | No |
| TCGA-BP-5192 | 59 | MALE | 2 | 1 | No |
| TCGA-BP-5194 | 39 | MALE | 0 | 2 | No |
| TCGA-BP-5195 | 75 | MALE | 0 | 0 | Yes |
| TCGA-BP-5196 | 53 | MALE | 0 | 2 | Yes |
| TCGA-BP-5198 | 72 | MALE | 1 | 2 | Yes |
| TCGA-BP-5199 | 58 | MALE | 1 | 1 | No |
| TCGA-BP-5200 | 44 | MALE | 0 | 2 | No |
| TCGA-BP-5201 | 63 | MALE | 0 | 1 | Yes |
| TCGA-BP-5202 | 75 | MALE | 0 | 2 | Yes |
| TCGA-B8-4143 | 66 | FEMALE | 1 | 0 | Yes |
| TCGA-B8-4148 | 63 | FEMALE | 0 | 0 | Yes |
| TCGA-B8-4150 | 62 | MALE | 2 | 0 | Yes |
| TCGA-B8-4151 | 51 | FEMALE | 0 | 2 | Yes |
| TCGA-B8-4153 | 74 | MALE | 0 | 1 | Yes |
| TCGA-B8-4154 | 73 | FEMALE | 1 | 0 | No |
| TCGA-B8-4619 | 58 | MALE | 2 | 2 | No |
| TCGA-B8-4620 | 70 | FEMALE | 1 | 2 | Yes |
| TCGA-B8-4621 | 63 | MALE | 1 | 0 | Yes |
| TCGA-B8-4622 | 57 | MALE | 0 | 0 | Yes |
| TCGA-B8-5158 | 56 | MALE | 2 | 2 | Yes |
| TCGA-B8-5159 | 61 | FEMALE | 0 | 2 | Yes |
| TCGA-B8-5162 | 63 | MALE | 0 | 1 | No |
| TCGA-B8-5163 | 63 | FEMALE | 0 | 2 | Yes |
| TCGA-B8-5164 | 65 | MALE | 2 | 1 | No |
| TCGA-B8-5165 | 43 | MALE | 0 | 1 | Yes |
| TCGA-B8-5545 | 42 | MALE | 1 | 2 | Yes |
| TCGA-B8-5546 | 38 | FEMALE | 0 | 2 | Yes |
| TCGA-B8-5549 | 53 | MALE | 0 | 2 | Yes |
| TCGA-B8-5550 | 71 | MALE | 2 | 0 | Yes |
| TCGA-B8-5551 | 65 | FEMALE | 1 | 2 | Yes |
| TCGA-B8-5552 | 41 | FEMALE | 0 | 0 | No |
| TCGA-B8-5553 | 67 | FEMALE | 2 | 2 | Yes |
| TCGA-B8-6180 | 70 | FEMALE | 1 | 2 | Yes |
| TCGA-B8-6181 | 81 | MALE | 1 | 1 | Yes |
| TCGA-B8-6182 | 63 | FEMALE | 0 | 2 | Yes |
| TCGA-B8-6183 | 51 | FEMALE | 2 | 1 | Yes |
| TCGA-B8-6185 | 66 | MALE | 0 | 0 | Yes |
| TCGA-B0-4690 | 65 | MALE | 1 | 2 | Yes |
| TCGA-B0-4691 | 55 | MALE | 1 | 1 | No |
| TCGA-B0-4693 | 72 | FEMALE | 0 | 0 | Yes |
| TCGA-B0-4697 | 46 | FEMALE | 0 | 2 | Yes |
| TCGA-B0-4700 | 60 | MALE | 0 | 1 | Yes |
| TCGA-B0-4701 | 66 | FEMALE | 2 | 0 | Yes |
| TCGA-B0-4703 | 51 | MALE | 1 | 1 | No |
| TCGA-B0-4706 | 61 | MALE | 2 | 2 | Yes |
| TCGA-B0-4707 | 63 | MALE | 1 | 2 | Yes |
| TCGA-B0-4710 | 75 | FEMALE | 0 | 2 | Yes |
| TCGA-B0-4713 | 76 | FEMALE | 0 | 1 | Yes |
| TCGA-B0-4714 | 81 | MALE | 0 | 0 | Yes |
| TCGA-B0-4718 | 57 | MALE | 1 | 2 | Yes |
| TCGA-B0-4810 | 47 | MALE | 1 | 1 | No |
| TCGA-B0-4811 | 48 | MALE | 1 | 0 | No |
| TCGA-B0-4817 | 81 | MALE | 0 | 1 | Yes |
| TCGA-B0-4818 | 68 | FEMALE | 1 | 1 | Yes |
| TCGA-B0-4821 | 69 | FEMALE | 0 | 0 | Yes |
| TCGA-B0-4822 | 78 | MALE | 1 | 2 | No |
| TCGA-B0-4824 | 49 | FEMALE | 0 | 2 | Yes |
| TCGA-B0-4827 | 77 | FEMALE | 0 | 1 | Yes |
| TCGA-B0-4836 | 61 | MALE | 1 | 2 | Yes |
| TCGA-B0-4838 | 69 | FEMALE | 1 | 2 | Yes |
| TCGA-B0-4839 | 80 | FEMALE | 1 | 2 | Yes |
| TCGA-B0-4841 | 63 | MALE | 1 | 1 | Yes |
| TCGA-B0-4843 | 57 | MALE | 0 | 2 | Yes |
| TCGA-B0-4845 | 70 | MALE | 1 | 2 | Yes |
| TCGA-B0-4846 | 52 | MALE | 1 | 0 | No |
| TCGA-B0-4852 | 78 | FEMALE | 0 | 0 | Yes |
| TCGA-B0-5075 | 77 | FEMALE | 1 | 1 | Yes |
| TCGA-B0-5077 | 77 | MALE | 0 | 1 | Yes |
| TCGA-B0-5081 | 79 | FEMALE | 1 | 0 | No |
| TCGA-B0-5084 | 33 | MALE | 2 | 0 | No |
| TCGA-B0-5085 | 76 | FEMALE | 1 | 0 | Yes |
| TCGA-B0-5088 | 53 | MALE | 1 | 2 | Yes |
| TCGA-B0-5092 | 53 | FEMALE | 1 | 2 | Yes |
| TCGA-B0-5094 | 62 | MALE | 0 | 2 | Yes |
| TCGA-B0-5095 | 81 | MALE | 0 | 1 | Yes |
| TCGA-B0-5097 | 59 | FEMALE | 0 | 2 | Yes |
| TCGA-B0-5100 | 72 | MALE | 0 | 1 | Yes |
| TCGA-B0-5102 | 74 | FEMALE | 0 | 2 | No |
| TCGA-B0-5104 | 90 | FEMALE | 0 | 0 | Yes |
| TCGA-B0-5106 | 64 | MALE | 1 | 2 | Yes |
| TCGA-B0-5107 | 65 | FEMALE | 0 | 0 | Yes |
| TCGA-B0-5108 | 54 | MALE | 0 | 2 | No |
| TCGA-B0-5109 | 69 | MALE | 1 | 0 | No |
| TCGA-B0-5110 | 71 | FEMALE | 0 | 2 | Yes |
| TCGA-B0-5113 | 69 | FEMALE | 0 | 0 | Yes |
| TCGA-B0-5115 | 43 | MALE | 0 | 1 | No |
| TCGA-B0-5116 | 52 | MALE | 2 | 1 | No |
| TCGA-B0-5117 | 40 | MALE | 2 | 2 | No |
| TCGA-B0-5119 | 61 | FEMALE | 1 | 2 | Yes |
| TCGA-B0-5120 | 72 | FEMALE | 2 | 2 | No |
| TCGA-B0-5121 | 56 | MALE | 0 | 2 | Yes |
| TCGA-B0-5399 | 46 | MALE | 2 | 1 | No |
| TCGA-B0-5400 | 59 | FEMALE | 1 | 2 | Yes |
| TCGA-B0-5402 | 64 | MALE | 0 | 1 | Yes |
| TCGA-B0-5693 | 47 | MALE | 2 | 2 | Yes |
| TCGA-B0-5694 | 71 | MALE | 2 | 1 | Yes |
| TCGA-B0-5695 | 61 | FEMALE | 0 | 2 | Yes |
| TCGA-B0-5696 | 69 | MALE | 2 | 1 | No |
| TCGA-B0-5697 | 50 | MALE | 1 | 0 | No |
| TCGA-B0-5698 | 77 | MALE | 1 | 1 | Yes |
| TCGA-B0-5700 | 77 | MALE | 1 | 1 | Yes |
| TCGA-B0-5701 | 65 | MALE | 2 | 1 | Yes |
| TCGA-B0-5702 | 71 | MALE | 1 | 1 | Yes |
| TCGA-B0-5703 | 73 | MALE | 1 | 2 | Yes |
| TCGA-B0-5705 | 65 | FEMALE | 0 | 1 | Yes |
| TCGA-B0-5707 | 39 | FEMALE | 2 | 0 | Yes |
| TCGA-B0-5709 | 62 | FEMALE | 1 | 2 | Yes |
| TCGA-B0-5710 | 57 | MALE | 0 | 2 | Yes |
| TCGA-B0-5711 | 50 | MALE | 0 | 2 | Yes |
| TCGA-B0-5712 | 68 | FEMALE | 0 | 1 | Yes |
| TCGA-B0-5713 | 75 | FEMALE | 0 | 2 | Yes |
| TCGA-B0-5812 | 53 | MALE | 0 | 2 | Yes |
